# A Comparative Study of Early Maladaptive Schema Profiles in Individuals Convicted of Homicide and Individuals from the Community: The Moderating Role of Sex

**DOI:** 10.3390/bs16091535

**Published:** 2026-08-31

**Authors:** Risvan Vlad Rusu, Cristian Delcea

**Affiliations:** 1 Department of Research, Institute of Sexology, 400394 Cluj-Napoca, Romania; risvanvladrusu@sexology.ro; 2 Multidisciplinary Doctoral School, Vasile Goldiș Western University of Arad, 310025 Arad, Romania

**Keywords:** Early Maladaptive Schemas, Schema Therapy, homicide, violent offending, forensic psychology, sex differences, incarceration

## Abstract

**Background:** Early Maladaptive Schemas (EMSs) have been associated with violent and antisocial behavior, but little is known about current EMS profiles among individuals convicted of homicide or whether forensic community differences vary according to sex. This study examined the independent and interactive associations of incarceration status and sex with EMSs. **Methods:** This cross-sectional, postdictive study included 492 participants: 246 incarcerated individuals convicted of homicide and 246 community participants. EMSs were assessed using the Young Schema Questionnaire–Short Form 3 (YSQ-S3). A 2 × 2 factorial multivariate analysis of covariance examined the effects of incarceration status, sex, and their interaction across 18 EMSs while controlling for age and educational attainment. **Results:** Significant multivariate effects were found for incarceration status, Pillai’s Trace = 0.417, *F*(18, 469) = 18.65, *p* < 0.001; sex, Pillai’s Trace = 0.446, *F*(18, 469) = 20.94, *p* < 0.001; and their interaction, Pillai’s Trace = 0.411, *F*(18, 469) = 18.21, *p* < 0.001. After Bonferroni correction across the 18 outcomes, incarcerated participants reported higher Emotional Deprivation and Abandonment, whereas community participants scored higher on several schemas involving impaired autonomy, negative self-evaluation, emotional overcontrol, impaired limits, and self-regulation. Mistrust/Abuse did not remain a robust aggregate status effect. These marginal patterns were qualified by the interaction: forensic community differences varied substantially by sex, particularly for Failure, Entitlement/Grandiosity, and Insufficient Self-Control/Self-Discipline. **Conclusions:** Current EMS profiles were selective and sex-contingent rather than globally elevated among individuals convicted of homicide. Because EMSs were assessed after the offense, conviction, and incarceration, the findings are postdictive and do not establish that the identified schemas predicted or caused homicidal behavior. Schema-informed assessment may complement broader multimethod forensic formulation, but the findings do not establish treatment effectiveness.

## 1. Introduction

### 1.1. Early Maladaptive Schemas

î Early Maladaptive Schemas (EMSs) constitute the central construct of Schema Therapy, an integrative form of psychotherapy developed by Jeffrey Young to explain the developmental origins of enduring psychological dysfunction and maladaptive personality functioning (Young et al., 2003) Extending Beck’s cognitive theory, Schema Therapy conceptualizes EMSs as broad, pervasive cognitive–emotional patterns comprising memories, emotions, cognitions, and bodily sensations concerning oneself and interpersonal relationships. These patterns originate during childhood or adolescence, become elaborated throughout life, and are dysfunctional to a significant degree (Young et al., 2003; Oei & Baranoff, 2007). Unlike automatic thoughts or situation-specific dysfunctional beliefs, EMSs represent relatively stable psychological structures that influence how individuals interpret experiences, regulate emotions, and respond behaviorally across interpersonal contexts.

Young’s model identifies 18 EMSs organized into five higher-order domains: disconnection and rejection, impaired autonomy and performance, impaired limits, other-directedness, and overvigilance and inhibition. These domains reflect the chronic frustration of five universal emotional needs—namely, secure attachment, autonomy and competence, realistic limits, freedom to express emotions, and spontaneity—and provide a developmental framework for understanding enduring patterns of psychological vulnerability (Young et al., 2003). Recent systematic reviews further support the clinical relevance of this hierarchical organization, demonstrating that these schema domains capture meaningful differences in cognitive vulnerability across both clinical and community populations (Bär et al., 2023).

A defining feature of Schema Therapy is its developmental perspective, which assumes that EMSs emerge through the interaction between individual temperament and chronically adverse interpersonal experiences rather than isolated traumatic events (Young et al., 2003; Láng, 2015). Repeated emotional deprivation, abuse, neglect, rejection, excessive criticism, overprotection, overindulgence, and exposure to maladaptive parental attitudes are considered major developmental pathways leading to schema formation. These assumptions have received substantial empirical support. In a systematic review of 34 empirical studies, Aloi et al. (2025a) concluded that EMSs consistently mediate the relationship between childhood maltreatment and adult psychopathology, particularly through the disconnection and rejection and impaired autonomy and performance domains. Likewise, Bär et al. (2023) identified adverse developmental experiences as one of the strongest predictors of maladaptive schema formation across psychiatric disorders. Parenting practices also play a central role in schema development. Specifically, dysfunctional parenting practices, including overprotection, emotional neglect, and excessive control, have been associated with schemas belonging to the impaired autonomy and performance and disconnection and rejection domains (Basso et al., 2019). Collectively, these findings provide robust empirical support for Young’s developmental model and suggest that EMSs represent enduring cognitive–emotional adaptations through which adverse childhood experiences influence later psychological functioning.

Beyond their developmental origins, EMSs are increasingly recognized as transdiagnostic vulnerability factors contributing to a broad spectrum of psychological disorders and maladaptive interpersonal functioning (Aghababaei et al., 2026; Oromendia et al., 2023). According to Young’s Schema Therapy model, activated EMSs trigger maladaptive coping responses, including surrender, avoidance, and overcompensation, which temporarily reduce emotional distress while simultaneously reinforcing the underlying schemas (Young et al., 2003). Consistent with this framework, recent reviews indicate that elevated EMS endorsement is observed across depressive, anxiety, obsessive-compulsive, trauma-related, substance use, eating, and personality disorders, supporting the conceptualization of EMSs as common cognitive vulnerabilities rather than disorder-specific characteristics (Bär et al., 2023; Aloi et al., 2025b). Earlier meta-analytic evidence likewise demonstrated robust associations between EMSs and depressive symptoms, particularly for schemas within the Disconnection and Rejection domain (Hawke & Provencher, 2011). Beyond psychopathology, EMSs have consistently been associated with insecure attachment, interpersonal dysfunction, reduced satisfaction in romantic relationships, and broader impairments in social functioning (Thimm, 2013; Kover et al., 2024). Because EMSs influence how individuals perceive interpersonal experiences, regulate emotions, and respond to social challenges, they have increasingly been investigated as potential mechanisms underlying externalizing behaviors, including aggression and criminal offending. This growing body of evidence provides the theoretical foundation for examining EMSs within forensic populations.

In addition to EMSs and their five higher-order domains, Schema Therapy also describes schema modes, which represent the moment-to-moment emotional, cognitive, physiological, and behavioral states that emerge when one or more EMSs become activated. Unlike EMSs, which are relatively stable trait-like cognitive structures organized into five higher-order domains, schema modes are not classified into these domains. Rather, they reflect the dynamic expression of underlying EMSs together with the individual’s coping responses in specific situations (Young et al., 2003; Rafaeli et al., 2011).

### 1.2. Early Maladaptive Schemas and Criminal Behavior

A growing body of evidence suggests that the influence of EMSs extends beyond internalizing psychopathology to encompass aggression, violence, and criminal offending. Within Young’s Schema Therapy model, EMSs bias the interpretation of interpersonal experiences, increasing the likelihood that situations involving rejection, frustration, or perceived injustice will elicit intense negative emotions and maladaptive coping responses (Young et al., 2003). Consequently, persistent maladaptive schema activation may impair emotional regulation, promote hostile interpretations of social interactions, and increase the likelihood of externalizing behaviors. Rather than conceptualizing violent offending solely as the product of criminal attitudes or cognitive distortions, Schema Therapy views antisocial behavior as the behavioral manifestation of enduring cognitive–emotional vulnerabilities shaped by adverse developmental experiences. This perspective has stimulated growing interest in applying the Schema Therapy model within forensic psychology to better understand the psychological mechanisms underlying persistent offending (Beck, 2020; Soygüt et al., 2009).

Recent empirical evidence supports this theoretical framework. In a systematic review, Sousa et al. (2024) concluded that EMSs and maladaptive schema modes represent important therapeutic targets in forensic and correctional settings, with schema-focused interventions demonstrating promising effects on reducing maladaptive cognitions and dynamic risk factors associated with recidivism. Similarly, Turhan et al. (2024) reported that offenders consistently exhibit elevated EMSs, particularly within the disconnection and rejection and impaired limits domains, highlighting the role of interpersonal mistrust, emotional deprivation, entitlement, and deficient self-control in understanding criminal behavior. Experimental evidence further indicates that EMSs contribute to aggressive behavior indirectly through emotional dysregulation. For example, Van Wijk-Herbrink et al. (2021) demonstrated that the association between perceived injustice and aggressive behavior is mediated by state anger and is particularly pronounced among individuals with elevated abandonment and entitlement schemas. Collectively, these findings suggest that EMSs influence offending not only by shaping maladaptive beliefs but also by amplifying emotional and behavioral responses to interpersonal stressors.

Research conducted in incarcerated populations further indicates that offending is associated with specific identifiable schema profiles rather than generalized elevations across all EMSs. Forensic studies have consistently reported a higher endorsement of schemas related to interpersonal rejection, mistrust, emotional deprivation, impaired self-control, and entitlement, supporting the view that specific cognitive vulnerability patterns characterize offender populations (Oettingen et al., 2023). Despite these advances, several important limitations remain. Most existing studies have focused on highly specific offender groups, including violent, sexual, or juvenile offenders, whereas comparatively few have directly compared incarcerated individuals with community samples using the complete Young’s Schema Therapy model. Furthermore, the potential role of sex in shaping these associations has received limited empirical attention, as described in the next section (Smith & Davis, 2025).

### 1.3. Sex Differences in Early Maladaptive Schemas

Evidence accumulated over the past two decades suggests that sex influences the development and expression of Early Maladaptive Schemas (EMSs), although the magnitude and consistency of these differences vary across studies (Young et al., 2003; Shorey et al., 2012). Developmental models propose that biological predispositions interact with gender-specific socialization experiences and differential exposure to adverse childhood experiences, contributing to distinct schema profiles in men and women (Shorey et al., 2012). Across both community and clinical populations, women generally report higher endorsement of schemas related to emotional deprivation, abandonment, defectiveness/shame, social isolation, and self-sacrifice, whereas men more frequently endorse schemas associated with entitlement/grandiosity, insufficient self-control, and other dimensions reflecting impaired limits and externalizing tendencies (Shorey et al., 2012; Irkörücü, 2016). Together, these findings suggest that sex-related developmental experiences influence not only the severity of individual schemas but also the broader organization of cognitive vulnerability (Mahalik et al., 2003).

Evidence from forensic populations, however, remains comparatively limited and inconsistent. Existing studies indicate that female offenders tend to report greater endorsement of trauma-related and interpersonal schemas, whereas male offenders more frequently exhibit schemas associated with hostility, impaired limits, and externalizing behaviors (Dawes, 2014). Nevertheless, most forensic investigations have either examined men and women separately or treated sex solely as a descriptive variable, rather than evaluating whether it moderates the relationship between incarceration status and EMS profiles. Consequently, it remains unclear whether incarceration status is associated with similar EMS profiles in men and women or whether distinct sex-specific patterns characterize these populations. Addressing this gap may improve the understanding of cognitive vulnerabilities associated with criminal offending and support the development of more individualized schema-focused assessment and intervention strategies within forensic settings.

### 1.4. Sociodemographic Factors Associated with Early Maladaptive Schemas

Although EMSs are conceptualized as relatively stable cognitive-emotional structures, sociodemographic characteristics may contribute to variability in schema endorsement and should therefore be considered when comparing heterogeneous populations. Among sociodemographic variables, age has received the greatest empirical attention and has been the most consistently examined potential source of variation in EMS assessment. Although EMSs appear to remain relatively stable across adulthood, evidence suggests that the expression of specific schemas may vary modestly with age without compromising the structural validity of the construct. For example, Pauwels et al. (2014) demonstrated substantial age neutrality of the Young Schema Questionnaire while identifying limited age-related differences for several individual schemas. Likewise, Santos et al. (2018) confirmed the measurement invariance of the Young Schema Questionnaire across adolescent samples, supporting meaningful comparisons of EMSs across developmental stages.

Compared with age, the relationship between educational attainment and EMSs has received considerably less empirical attention. Nevertheless, educational attainment is routinely considered an important sociodemographic characteristic in studies involving both clinical and forensic populations because differences in educational background may influence questionnaire responses and represent a potential source of confounding when comparing heterogeneous groups. Accordingly, age and educational attainment were selected as covariates in the present study because they are the sociodemographic variables most consistently considered in the EMS literature and were also the variables on which the incarcerated and community groups differed. Controlling for these variables allowed the associations between incarceration status and EMS profiles to be examined independently of these potential sources of demographic confounding.

### 1.5. The Present Study

Despite the growing body of research linking EMSs to criminal behavior, several important gaps remain. First, relatively few studies have directly compared incarcerated individuals with community participants using the complete Young’s Schema Therapy model. Second, although evidence suggests that men and women differ in schema endorsement, the potential moderating role of sex in the association between incarceration status and EMS profiles has received limited empirical attention. Addressing these gaps may improve the understanding of the cognitive vulnerabilities associated with criminal offending and inform the development of more individualized assessment and intervention strategies within forensic settings.

Accordingly, this cross-sectional comparative study examined differences in EMS profiles between incarcerated individuals convicted of homicide and community participants, while simultaneously investigating the effects of sex and the interaction between incarceration status and sex. Age and educational attainment were included as covariates to reduce the potential influence of sociodemographic differences between groups.

Based on the available theoretical and empirical evidence, the following hypotheses were formulated. Specifically, the first hypothesis focused on the *Disconnection and Rejection* and *Impaired Limits* domains because previous research has consistently linked these domains with insecure attachment, interpersonal mistrust, emotional deprivation, impulsivity, and deficient behavioral regulation, characteristics commonly observed in violent offending (Young et al., 2003; Van Wijk-Herbrink et al., 2021; Turhan et al., 2024).

**H1.**  *Individuals incarcerated for homicide convictions will exhibit a distinct profile of Early Maladaptive Schemas compared with community participants, characterized by higher levels of schemas primarily related to the disconnection and rejection and impaired limits domains*.

**H2.**  *Men and women will exhibit distinct profiles of Early Maladaptive Schemas, reflecting sex-related differences in schema development and interpersonal functioning*.

**H3.**  *The profile of Early Maladaptive Schemas associated with incarceration status will differ according to sex, indicating that sex moderates the relationship between incarceration status and Early Maladaptive Schemas*.

## 2. Materials and Methods

### 2.1. Study Design and Participants

This study employed a cross-sectional, postdictive, 2 × 2 factorial group-comparison design to examine current EMS profiles as a function of incarceration status (incarcerated participants convicted of homicide vs. community participants), sex, and their interaction, while controlling for age and educational attainment. Because EMSs were assessed after the index offense, conviction, and incarceration, the study was postdictive rather than predictive: it examined whether current EMS profiles differed between groups defined by a previously occurring outcome but could not establish whether the observed schemas preceded or predicted homicidal behavior. The study was conducted in accordance with the ethical principles of the Declaration of Helsinki and is reported in accordance with the Strengthening the Reporting of Observational Studies in Epidemiology (STROBE) guidelines.

Participants were recruited as part of a broader research project examining psychological and cognitive factors associated with homicidal behavior. A purposive, sex-stratified recruitment strategy was used to obtain equal numbers of men and women within both the incarcerated and community groups. This sampling strategy was selected to provide adequate and balanced statistical power for examining the incarceration status × sex interaction. Accordingly, the resulting sex distribution was determined by the study design and was not intended to reproduce the substantially unequal distribution of men and women in the broader population of individuals convicted of homicide.

Incarcerated participants were recruited from several correctional institutions located in different regions of Romania, following authorization from the Romanian National Administration of Penitentiaries. Eligibility criteria included being between 18 and 90 years of age, having received a final conviction for a homicide offense, having completed at least eight years of formal education, possessing sufficient cognitive capacity to understand and complete the assessment battery, and providing written informed consent. A total of 400 incarcerated individuals were initially considered for participation. After applying the eligibility criteria and excluding incomplete or invalid assessment protocols, 246 participants were retained in the final incarcerated sample, comprising 123 men and 123 women.

Community participants were recruited from several Romanian localities through university centers, university communication channels, online announcements, and social media platforms. Eligibility criteria included being between 18 and 90 years of age, having completed at least eight years of formal education, having no prior criminal record, no previous criminal conviction, and no history of incarceration, and providing written informed consent. Thus, eligibility was not limited to the absence of a homicide conviction; individuals reporting any previous criminal conviction were excluded from the community sample. A total of 510 community participants were initially considered. Following application of the eligibility criteria and exclusion of incomplete or invalid assessment protocols, 246 participants were retained, comprising 123 men and 123 women.

The final analytical sample therefore included 492 participants distributed equally across the four study cells: 123 incarcerated men, 123 incarcerated women, 123 community men, and 123 community women. In the incarcerated group, the mean age was 37.33 years (SD = 9.94) and the mean educational attainment was 10.93 years (SD = 1.82). In the community group, the mean age was 30.96 years (SD = 10.42) and the mean educational attainment was 11.63 years (SD = 2.13). Detailed demographic characteristics, as well as offense-related, sentencing, victim, and criminal-history characteristics of the incarcerated group, are reported in Section 3.1.

### 2.2. Study Procedure

Before data collection, all eligible participants received written and verbal information about the purpose of the study, the voluntary nature of participation, the confidentiality of the collected data, and their right to withdraw from participation at any time without adverse consequences. Written informed consent was obtained from all participants before the assessment. Questionnaires were administered individually in a standardized format under the supervision of trained researchers, who were available to clarify the instructions when necessary. Participants were identified through non-identifying study codes, and no directly identifiable personal information was included in the analytical dataset.

The present investigation was conducted as part of a broader research project examining psychological and cognitive factors associated with homicidal behavior. The broader assessment battery included the Young Schema Questionnaire–Short Form 3 (YSQ-S3), the Eysenck Personality Questionnaire–Revised (EPQ-R), administered and scored through the Cognitrom computerized assessment platform, and the Cognitrom Analytical Reasoning Questionnaire (ARQ). The EPQ-R generated dimensional scores for Psychoticism, Extraversion, Neuroticism, Lie/response-presentation tendencies, Addiction, and Criminality, whereas the ARQ assessed analytical reasoning abilities. The primary hypothesis-testing analyses reported in the present study focused on EMSs. Consequently, the EPQ-R and ARQ scores were not included in the primary model; however, the EPQ-R Lie score was subsequently included as an additional covariate in a sensitivity analysis examining the robustness of the findings to measured response-presentation tendencies.

### 2.3. Measures

#### 2.3.1. Demographic Questionnaire

Participants completed a brief demographic questionnaire developed for the present study. The questionnaire collected information on age, sex, educational attainment, and incarceration status. Age and educational attainment were included as covariates in the statistical analyses, whereas sex and incarceration status were entered as the principal fixed factors.

#### 2.3.2. Young Schema Questionnaire–Short Form 3 (YSQ-S3)

EMSs were assessed using the Young Schema Questionnaire–Short Form 3 (YSQ-S3; Young, 2005). The instrument consists of 90 items assessing 18 EMSs, with five items measuring each schema. Responses are recorded on a 6-point Likert scale ranging from 1 (*Completely untrue of me*) to 6 (*Describes me perfectly*), with higher scores indicating stronger endorsement of the corresponding schema.

The YSQ-S3 assesses Emotional Deprivation, Abandonment, Mistrust/Abuse, Social Isolation, Defectiveness/Shame, Failure, Dependence/Incompetence, Vulnerability to Harm or Illness, Enmeshment/Undeveloped Self, Subjugation, Self-Sacrifice, Emotional Inhibition, Unrelenting Standards, Entitlement/Grandiosity, Insufficient Self-Control/Self-Discipline, Approval Seeking, Negativity/Pessimism, and Punitiveness. These schemas are organized into the five higher-order schema domains proposed by Young et al. (2003).

The YSQ-S3 protocols were administered and scored using the Cognitrom computerized assessment platform. After questionnaire completion, the software automatically processed the item responses and generated a T score for each of the 18 schemas. These scores were not calculated or transformed manually by the authors. The schema-level T-score outputs generated by the Cognitrom system were exported directly and used as the dependent variables in all subsequent statistical analyses. Accordingly, the descriptive and inferential results are expressed in the Cognitrom-generated T-score metric, with higher values indicating stronger relative endorsement of the corresponding schema. The covariate-adjusted estimated marginal means reported in the Results section therefore remain expressed in this metric and should not be interpreted as raw mean item ratings on the original 1–6 response scale.

The YSQ-S3 is widely used for assessing EMSs and has demonstrated satisfactory internal consistency, construct validity, and factorial validity across clinical and community populations (Kriston et al., 2012; Pauwels et al., 2014). In the present study, internal consistency was estimated using Cronbach’s alpha and indicated excellent reliability for the overall questionnaire (α = 0.93). Reliability coefficients for the five higher-order schema domains are presented in Table 1.

#### 2.3.3. Eysenck Personality Questionnaire–Revised (EPQ-R)

As part of the broader assessment protocol, participants completed the Eysenck Personality Questionnaire–Revised (EPQ-R; Eysenck et al., 1985), administered and scored through the Cognitrom computerized assessment platform. The platform generated scores for the Psychoticism, Extraversion, Neuroticism, and Lie dimensions, as well as the supplementary Addiction and Criminality dimensions. The primary analyses in the present study focused on EMSs; consequently, the EPQ-R personality dimensions were not included in the primary hypothesis-testing model.

The EPQ-R Lie score was used as an additional covariate in a sensitivity analysis. This dimension was treated as an indicator of socially desirable or defensive response-presentation tendencies, with higher scores reflecting a greater tendency toward favorable self-presentation. Its inclusion allowed us to examine whether the multivariate effects of incarceration status, sex, and their interaction remained stable after adjustment for measured response style. The Lie score was not interpreted as a comprehensive measure of common-method variance or as a complete correction for self-report bias. Furthermore, EPQ-R Psychoticism is conceptually distinct from psychopathy and from psychotic disorders and was not used as a proxy for either construct.

### 2.4. Statistical Analysis

Statistical analyses were performed using IBM SPSS Statistics Version 30 (IBM Corp., Armonk, NY, USA). Before hypothesis testing, the data were screened for missing values, data-entry errors, univariate and multivariate outliers, and the assumptions underlying multivariate analysis. Mahalanobis distances were calculated across the 18 EMS variables to identify multivariate outliers. Using the conservative chi-square criterion of χ^2^(18) = 42.31, *p* < 0.001, 19 observations exceeded the critical value. Inspection of these cases indicated that their values remained within the observed score ranges and represented plausible multivariate response profiles rather than identifiable data-entry errors. Consequently, all observations were retained for the subsequent analyses. Multicollinearity was also evaluated using tolerance and variance inflation factor (VIF) statistics, which indicated no evidence of problematic collinearity among the covariates.

Descriptive statistics were calculated for the demographic variables and study measures. Group differences in demographic characteristics were examined using independent-samples *t*-tests for continuous variables and chi-square tests for categorical variables.

To examine the study hypotheses, a 2 × 2 multivariate analysis of covariance (MANCOVA) was conducted, with incarceration status (incarcerated vs. community) and sex (men vs. women) entered as fixed factors. Age and educational attainment were included as covariates to account for demographic differences between the groups, and the 18 EMS scores served as the dependent variables. Multivariate effects were evaluated using Pillai’s Trace because of its relative robustness to moderate departures from multivariate normality and homogeneity of covariance matrices.

To examine the robustness of the findings to socially desirable or defensive response presentation, the factorial MANCOVA was repeated as a sensitivity analysis with the EPQ-R Lie score included as an additional covariate. The Lie score was treated as a measured indicator of response-presentation tendencies rather than as a comprehensive statistical correction for common-method variance. This analysis examined whether the multivariate effects of incarceration status, sex, and their interaction remained stable after accounting for individual differences in measured response style.

Significant multivariate effects in the primary model were followed by univariate analyses of covariance (ANCOVAs) for each EMS. Bonferroni-adjusted pairwise comparisons were performed where appropriate to account for multiple comparisons. Effect sizes are reported as partial eta squared (ηp^2^). To obtain robust estimates of parameter precision, 95% bias-corrected and accelerated (BCa) bootstrap confidence intervals were computed using 1000 bootstrap resamples.

Statistical significance was established at *p* < 0.05 for all analyses.

## 3. Results

### 3.1. Descriptive Statistics

The demographic characteristics of the study sample are presented in Table 2. The incarcerated and community groups each comprised 246 participants and were balanced with respect to sex (50% male, 50% female). Participants ranged in age from 18 to 90 years. î Independent-samples *t*-tests indicated that the incarcerated group (M = 37.95, SD = 9.31) was significantly older than the community group (M = 32.95, SD = 10.59), *t*(244) = 3.93, *p* < 0.001, Cohen’s *d* = 0.50, indicating a medium effect size. Conversely, the community group (M = 11.84, SD = 2.24) had significantly higher educational attainment than the incarcerated group (M = 10.94, SD = 1.71), *t*(244) = −3.55, *p* < 0.001, Cohen’s *d* = 0.45, also representing a medium effect size.

To provide a more comprehensive description of the forensic sample, the incarcerated subgroup was further characterized in terms of age at the index offense, functional classification of the index offense, victim–offender relationship, number of victims, sentence characteristics, time served at assessment, and recorded criminal history. These characteristics are reported for the incarcerated sample as a whole and separately for men and women in Table 3.

### 3.2. Preliminary Analyses

Prior to the main analyses, the assumptions underlying multivariate analysis were examined. Multivariate outliers were identified using the Mahalanobis distance; inspection of these cases did not reveal data entry errors or values outside the permissible ranges of the study variables, and so all observations were retained for subsequent analyses. Multicollinearity diagnostics indicated no evidence of collinearity between the covariates (all VIFs = 1.00). Box’s *M* test was statistically significant (*Box’s M* = 4019.51, *F*(513, 501,187.34) = 7.33, *p* < 0.001), indicating heterogeneity of covariance matrices. Therefore, Pillai’s Trace, which is considered the most robust multivariate test statistic under violations of this assumption (Olson, 1974), was used to evaluate multivariate effects. Levene’s tests indicated unequal error variances for several dependent variables; therefore, the multivariate findings were interpreted primarily on the basis of Pillai’s Trace and supported by bias-corrected and accelerated (BCa) bootstrap confidence intervals based on 1000 stratified bootstrap samples.

### 3.3. Multivariate Effects

To examine the study hypotheses, a 2 × 2 multivariate analysis of covariance (MANCOVA) was conducted with incarceration status (incarcerated vs. community) and sex (men vs. women) entered as fixed factors. Age and educational attainment were included as covariates, and the 18 EMSs served as the dependent variables.

The multivariate effects are presented in Table 4.

The analysis revealed statistically significant multivariate effects of age (Pillai’s Trace = 0.062, *F*(18, 469) = 1.71, *p* = 0.035, ηp^2^ = 0.062) and educational attainment (Pillai’s Trace = 0.065, *F*(18, 469) = 1.81, *p* = 0.022, ηp^2^ = 0.065). Significant multivariate main effects were also observed for incarceration status (Pillai’s Trace = 0.417, *F*(18, 469) = 18.65, *p* < 0.001, ηp^2^ = 0.417) and sex (Pillai’s Trace = 0.446, *F*(18, 469) = 20.94, *p* < 0.001, ηp^2^ = 0.446). In addition, the incarceration status × sex interaction was statistically significant (Pillai’s Trace = 0.411, *F*(18, 469) = 18.21, *p* < 0.001, ηp^2^ = 0.411), indicating that the overall EMS profile varied as a function of both incarceration status and sex after controlling for age and educational attainment.

As a sensitivity analysis, the factorial MANCOVA was repeated with the EPQ-R Lie score included as an additional covariate. The multivariate contribution of the EPQ-R Lie score was not statistically significant, Pillai’s Trace = 0.056, *F*(18, 468) = 1.54, *p* = 0.071, ηp^2^ = 0.056. After adjustment for measured response-presentation tendencies, the multivariate effects of incarceration status, Pillai’s Trace = 0.421, *F*(18, 468) = 18.90, *p* < 0.001, ηp^2^ = 0.421, sex, Pillai’s Trace = 0.452, *F*(18, 468) = 21.42, *p* < 0.001, ηp^2^ = 0.452, and the incarceration status × sex interaction, Pillai’s Trace = 0.413, *F*(18, 468) = 18.32, *p* < 0.001, ηp^2^ = 0.413, remained statistically significant. These effects were substantively similar to those obtained in the primary model, indicating that the principal multivariate findings were not materially altered after statistical adjustment for the measured response-presentation tendency.

To identify the individual EMSs contributing to the significant multivariate effects, the univariate tests associated with the MANCOVA were examined. Bonferroni-adjusted estimated marginal means are presented in Table 5, and the complete results of the univariate tests are reported in Supplementary Table S1.

### 3.4. Marginal Main Effect of Incarceration Status (Hypothesis 1)

Hypothesis 1 predicted that individuals convicted of homicide would exhibit a distinct EMS profile characterized by higher endorsement of schemas primarily belonging to the Disconnection/Rejection and Impaired Limits domains.

The MANCOVA revealed a statistically significant multivariate effect of incarceration status, indicating that the overall EMS profile differed between incarcerated and community participants after controlling for age and educational attainment (see Table 4). Follow-up univariate ANCOVAs were evaluated using a Bonferroni correction across the 18 EMS outcomes. After this familywise correction, significant effects of incarceration status remained for Emotional Deprivation, Abandonment, Defectiveness/Shame, Failure, Dependence/Incompetence, Vulnerability to Harm or Illness, Enmeshment/Undeveloped Self, Subjugation, Self-Sacrifice, Emotional Inhibition, Unrelenting Standards, Entitlement/Grandiosity, Insufficient Self-Control/Self-Discipline, Approval Seeking, and Negativity/Pessimism. No significant corrected effects were found for Mistrust/Abuse, Social Isolation, or Punitiveness. Although the unadjusted effect for Mistrust/Abuse reached the conventional significance threshold (*p* = 0.036), it did not survive correction across the 18 outcomes (Bonferroni-adjusted *p* = 0.649) and was therefore not interpreted as a robust status effect. Complete univariate statistics, including unadjusted and Bonferroni-adjusted *p* values, partial η^2^ values, and their 95% confidence intervals, are presented in Supplementary Table S1.

The directions of the effects that remained significant after the across-outcome correction were examined using the estimated marginal means. Individuals convicted of homicide reported higher adjusted scores on Emotional Deprivation and Abandonment than community participants. Conversely, community participants obtained higher adjusted scores on Defectiveness/Shame, Failure, Dependence/Incompetence, Vulnerability to Harm or Illness, Enmeshment/Undeveloped Self, Subjugation, Self-Sacrifice, Emotional Inhibition, Unrelenting Standards, Entitlement/Grandiosity, Insufficient Self-Control/Self-Discipline, Approval Seeking, and Negativity/Pessimism. No robust aggregate group differences were identified for Mistrust/Abuse, Social Isolation, or Punitiveness.

Overall, incarceration status was associated with a selective EMS profile rather than with uniformly elevated schema endorsement. Hypothesis 1 was only partially supported. The prediction concerning the Disconnection/Rejection domain received limited support because individuals convicted of homicide scored higher on Emotional Deprivation and Abandonment, but the pattern did not extend reliably to Mistrust/Abuse or Social Isolation after correction for multiple outcomes. The prediction concerning the Impaired Limits domain was not supported. Both Entitlement/Grandiosity and Insufficient Self-Control/Self-Discipline were significantly higher among community participants, contrary to the hypothesized direction.

These marginal status effects must nevertheless be interpreted in conjunction with the significant incarceration status × sex interaction reported in the subsequent analysis. In particular, the higher Entitlement/Grandiosity and Insufficient Self-Control/Self-Discipline scores observed among community participants were concentrated among men and did not represent a uniform difference across both sexes. Consequently, the main effect of incarceration status describes an average contrast across the sample and should not be generalized as a common EMS pattern applying equally to incarcerated women and men.

### 3.5. Marginal Main Effect of Sex (Hypothesis 2)

Hypothesis 2 predicted that women and men would exhibit distinct EMS profiles.

The MANCOVA revealed a statistically significant multivariate main effect of sex, Pillai’s Trace = 0.446, *F*(18, 469) = 20.94, *p* < 0.001, ηp^2^ = 0.446, indicating that the overall EMS profile differed between women and men after controlling for age and educational attainment (see Table 4). Follow-up univariate ANCOVAs were evaluated using a Bonferroni correction across the 18 EMS outcomes. After this familywise correction, significant sex effects remained for Emotional Deprivation, Abandonment, Mistrust/Abuse, Defectiveness/Shame, Failure, Dependence/Incompetence, Vulnerability to Harm or Illness, Enmeshment/Undeveloped Self, Subjugation, Self-Sacrifice, Emotional Inhibition, Unrelenting Standards, Entitlement/Grandiosity, Insufficient Self-Control/Self-Discipline, and Negativity/Pessimism. No significant corrected sex effects were found for Social Isolation, Approval Seeking, or Punitiveness. Although the unadjusted effect for Punitiveness reached the conventional significance threshold (*p* = 0.016), it did not survive correction across the 18 outcomes (Bonferroni-adjusted *p* = 0.285) and was therefore not interpreted as a robust sex effect. Complete univariate statistics, including unadjusted and Bonferroni-adjusted *p* values, partial η^2^ values, and their 95% confidence intervals, are presented in Supplementary Table S1.

The directions of the effects that remained significant after the across-outcome correction were examined using the estimated marginal means. Women obtained higher adjusted scores than men on Emotional Deprivation, Abandonment, and Mistrust/Abuse. Conversely, men obtained higher adjusted scores than women on Defectiveness/Shame, Failure, Dependence/Incompetence, Vulnerability to Harm or Illness, Enmeshment/Undeveloped Self, Subjugation, Self-Sacrifice, Emotional Inhibition, Unrelenting Standards, Entitlement/Grandiosity, Insufficient Self-Control/Self-Discipline, and Negativity/Pessimism. No robust aggregate sex differences were identified for Social Isolation, Approval Seeking, or Punitiveness.

Overall, these findings indicate that women and men differed in the configuration of their EMS profiles rather than simply in their overall level of schema endorsement. Women reported higher scores on selected schemas involving emotional deprivation, relational instability, and interpersonal mistrust, whereas men reported higher scores across several schemas involving impaired autonomy, negative self-evaluation, emotional overcontrol, and self-regulation. Hypothesis 2 was therefore supported at the multivariate profile level.

This marginal sex effect must nevertheless be interpreted in conjunction with the significant incarceration status × sex interaction reported below. The aggregate comparison does not demonstrate that each sex difference occurred in the same direction or with the same magnitude within both the incarcerated and community groups. Accordingly, Hypothesis 2 supports the presence of an overall sex-related difference in EMS configuration but not a universal psychological distinction applying uniformly across incarceration-status groups.

### 3.6. Interaction Between Incarceration Status and Sex (Hypothesis 3)

Hypothesis 3 predicted that sex would moderate the association between incarceration status and EMS profiles.

The MANCOVA revealed a statistically significant incarceration status × sex interaction, Pillai’s Trace = 0.411, *F*(18, 469) = 18.21, *p* < 0.001, ηp^2^ = 0.411, indicating that the association between incarceration status and the overall EMS profile differed between women and men after controlling for age and educational attainment (see Table 4). After Bonferroni correction across the 18 outcome-specific ANCOVAs, significant interaction effects remained for Emotional Deprivation, Social Isolation, Defectiveness/Shame, Failure, Dependence/Incompetence, Vulnerability to Harm or Illness, Enmeshment/Undeveloped Self, Subjugation, Self-Sacrifice, Emotional Inhibition, Unrelenting Standards, Entitlement/Grandiosity, Insufficient Self-Control/Self-Discipline, Approval Seeking, Negativity/Pessimism, and Punitiveness. The corrected interaction effect for Emotional Deprivation remained significant at Bonferroni-adjusted *p* = 0.032; the remaining significant interaction effects had Bonferroni-adjusted *p* values below 0.001. No significant interaction effects were found for Abandonment or Mistrust/Abuse. Complete univariate statistics are presented in Supplementary Table S1.

To characterize the significant interactions, estimated marginal means were examined using Bonferroni-adjusted factor-level comparisons within each EMS. Among men, incarcerated participants obtained higher adjusted scores than community participants on Emotional Deprivation. In contrast, community men obtained higher adjusted scores than incarcerated men on Social Isolation, Defectiveness/Shame, Failure, Dependence/Incompetence, Vulnerability to Harm or Illness, Enmeshment/Undeveloped Self, Subjugation, Self-Sacrifice, Emotional Inhibition, Unrelenting Standards, Entitlement/Grandiosity, Insufficient Self-Control/Self-Discipline, Approval Seeking, Negativity/Pessimism, and Punitiveness.

Among women, incarcerated participants obtained higher adjusted scores than community participants on Emotional Deprivation, Social Isolation, Defectiveness/Shame, Failure, Approval Seeking, Negativity/Pessimism, and Punitiveness. No significant incarceration-status differences among women were identified for Dependence/Incompetence, Vulnerability to Harm or Illness, Enmeshment/Undeveloped Self, Subjugation, Self-Sacrifice, Emotional Inhibition, Unrelenting Standards, Entitlement/Grandiosity, or Insufficient Self-Control/Self-Discipline.

Abandonment and Mistrust/Abuse were not included in the interpretation of the simple interaction pattern because their omnibus incarceration status × sex effects were not statistically significant. Any subgroup differences observed for these schemas therefore should not be interpreted as evidence that their associations with incarceration status differed according to sex.

Overall, the findings support Hypothesis 3. The association between incarceration status and EMSs was not uniform across women and men. Among men, the incarcerated group showed higher Emotional Deprivation, whereas community men showed higher scores across multiple schemas involving impaired autonomy, negative self-evaluation, emotional overcontrol, impaired limits, and self-regulation. Among women, the incarcerated group showed higher scores on a smaller but distinct set of interpersonal, self-evaluative, approval-related, pessimistic, and punitive schemas. The significant interactions therefore indicate that the EMS configuration associated with incarceration status differed substantially according to sex. These results establish statistical moderation within the present postdictive group comparison but do not identify the developmental or causal mechanisms responsible for the subgroup differences.

## 4. Discussion

### 4.1. Main Findings

The principal finding of the present study was the significant interaction between incarceration status and sex in the multivariate profile of Early Maladaptive Schemas (EMSs), after controlling for age and educational attainment. This interaction qualifies the main effects of incarceration status and sex: neither effect should be interpreted as a uniform difference applying equally across all four subgroups. More broadly, the findings did not reveal a generalized elevation of EMSs among individuals convicted of homicide. Instead, they indicated selective and sex-contingent configurations of schema endorsement.

At the aggregate level, the univariate effects that remained significant after Bonferroni correction across the 18 EMS outcomes indicated comparatively higher Emotional Deprivation and Abandonment among individuals convicted of homicide, whereas community participants scored higher on several schemas involving impaired autonomy, negative self-evaluation, emotional overcontrol, and self-regulation. The status effect for Mistrust/Abuse did not remain statistically significant after this familywise correction and is therefore not interpreted as a robust aggregate group difference. More importantly, the significant interactions demonstrated that the marginal status effects concealed markedly different subgroup patterns. Among men, the incarcerated group was distinguished principally by higher Emotional Deprivation and Abandonment, whereas community men obtained higher scores across a broader range of other schemas. Among women, the incarcerated group displayed a different and comparatively broader configuration spanning several attachment-related, interpersonal, self-evaluative, approval-related, negative-affect, and punitive themes, although the robustness of individual subgroup contrasts varied. Thus, membership in the homicide-convicted group was associated with different EMS configurations in men and women.

Against this background, H1 received only partial support. The prediction that participants convicted of homicide would show greater endorsement of schemas within the Disconnection/Rejection domain was supported for Emotional Deprivation and Abandonment, but not uniformly across the domain: Social Isolation did not show a significant aggregate status effect, and the status effect for Mistrust/Abuse did not survive correction across the 18 outcomes. The predicted elevation of the Impaired Limits domain was not supported. Entitlement/Grandiosity and Insufficient Self-Control/Self-Discipline were higher among community participants at the aggregate level, with the interaction indicating that these differences were concentrated among men. These findings therefore contradict the Impaired Limits component of H1 and demonstrate that the hypothesis cannot be evaluated adequately without considering the status × sex interaction.

This pattern is consistent with a dimensional and heterogeneous account of cognitive–emotional vulnerability in forensic populations. It also cautions against interpreting the number or magnitude of self-reported EMS elevations as a direct index of criminogenic risk or offense severity. Because EMSs were assessed after the index offense, conviction, and incarceration, the findings characterize current postdictive group differences rather than antecedents or predictors of homicidal behavior.

### 4.2. Interpreting the Incarceration Status × Sex Interaction

The interaction provides a more informative account of the data than either main effect considered alone. Among men, the contrast between higher Emotional Deprivation and Abandonment in the incarcerated group and broader elevations among community participants is incompatible with a simple deficit model in which men convicted of homicide necessarily report greater schema pathology across domains. Attachment-related expectations of emotional unavailability and relational instability may be particularly salient within the incarcerated male subgroup. Conversely, the comparatively lower endorsement of other EMSs should not be equated with lower psychological vulnerability or lower criminogenic risk, because a self-reported schema score does not directly measure offense-proximal emotional states, coping responses, behavioral regulation, or violent propensity.

The pattern among women was substantially different. Women convicted of homicide displayed a broader configuration of differences spanning attachment insecurity, interpersonal disconnection, negative self-evaluation, approval-related concerns, pessimism, and punitiveness. This pattern indicates that the overall incarceration-status effect comprised different configurations in women and men rather than a common offender profile. It also reinforces the importance of including women in forensic-schema research rather than extrapolating from predominantly male samples.

These subgroup differences do not, however, establish distinct developmental or causal pathways by sex. The present study did not assess gendered socialization, attachment style, childhood adversity, differential emotional development, coping styles, or offense-proximal psychological states. Interpretations invoking these processes should therefore be regarded as theoretically informed hypotheses rather than empirically supported explanations of the observed interaction. Psychiatric symptoms, personality characteristics, offense circumstances, institutional experiences, and other unmeasured variables may also contribute to the subgroup patterns.

The overall main effect of sex should be interpreted within the same boundary. Average differences between men and women describe the sample as a whole but do not demonstrate that the same sex difference was present within both incarceration groups. Moreover, the univariate sex effect for Punitiveness did not remain statistically significant after correction across the 18 EMS outcomes. Accordingly, the present findings support conclusions about sex-contingent EMS configurations, not essential, universal, or causally determined psychological differences between women and men.

### 4.3. EMS Profiles in Relation to Broader Forensic Personality Research

The apparently counterintuitive findings should first be interpreted in light of the significant incarceration status × sex interaction rather than solely from the marginal main effects of incarceration status. For Failure, Entitlement/Grandiosity, and Insufficient Self-Control/Self-Discipline, community men scored substantially higher than incarcerated men. However, women showed no significant incarceration-status differences in Entitlement/Grandiosity or Insufficient Self-Control/Self-Discipline, whereas the direction of the Failure contrast was reversed, with incarcerated women scoring higher than community women. Accordingly, the aggregate status effects do not indicate that community participants as a whole were more impaired on these schemas. Instead, they primarily reflect pronounced, sex-contingent differences, particularly the contrast between incarcerated and community men. The same interpretive caution applies to the other schemas for which community participants obtained higher marginal means, because most of these effects were also qualified by significant incarceration status × sex interactions.

The higher scores obtained by community men on the Impaired Limits schemas are not without precedent. Oettingen et al. (2024), using the YSQ-S3 in a sample of 102 incarcerated men convicted of sexual offenses and 167 non-criminal community men matched on age and educational attainment, similarly found that the community group scored significantly higher on the Impaired Limits domain, including Entitlement/Grandiosity and Insufficient Self-Control/Self-Discipline. Thus, the direction of the present male-specific findings for these two schemas has been observed in another forensic community comparison. However, Oettingen et al. (2024) found higher Failure scores among offenders rather than controls. This divergence underscores that EMS profiles may vary according to sex, offense type, clinical characteristics, correctional context, and sampling procedures and should not be treated as a monotonic indicator of criminal severity or psychological dysfunction.

A further consideration is that the YSQ-S3 assesses consciously endorsed, relatively enduring cognitive–emotional themes at the time of assessment and does not directly measure the situational schema modes or coping responses activated during an offense. Dunne et al. (2018), in a sample of 208 adult male prisoners, found that EMSs were associated with aggression but did not explain unique variance after schema modes were considered; instead, the Enraged Child, Impulsive Child, and Bully and Attack modes emerged as significant predictors of aggression. Similarly, Keulen-de Vos et al. (2016) found that vulnerability, abandonment, and loneliness-related modes were more evident in the period preceding criminal behavior, whereas impulsive, angry, and aggressive overcompensatory modes became more prominent during the offense. These findings suggest that lower explicit endorsement of Entitlement/Grandiosity or Insufficient Self-Control/Self-Discipline cannot be equated with the absence of entitlement-related, impulsive, or aggressive functioning in offense-proximal situations. Because schema modes and offense-proximal psychological states were not assessed in the present study, however, this interpretation represents a theoretically informed hypothesis rather than a mechanism demonstrated by the current data.

More broadly, the observed heterogeneity is consistent with research showing that forensic populations do not possess a single, uniform personality profile. Using the Temperament and Character Inventory in 148 young men convicted of violent or sexual offenses, Seidl et al. (2020) identified substantial variation in temperament and character dimensions and emphasized that offender characteristics and intervention-relevant capacities should be considered dimensionally rather than through a one-size-fits-all formulation. Their findings concerning self-directedness, cooperativeness, harm avoidance, and novelty seeking illustrate that personality-related functioning in violent offenders spans multiple domains and cannot be reduced to antisociality alone.

Evidence from a Romanian correctional context further supports a differentiated interpretation. Hurezan et al. (2024) compared incarcerated and community participants using Five-Factor Model dimensions and dark personality characteristics while controlling for age, sex, and impression management. Neuroticism, Extraversion, Openness, and selected dark characteristics contributed differently to distinctions between community participants, first-time offenders, and recidivists, whereas Agreeableness and Conscientiousness did not display the uniform effects that might be anticipated from a simple offender stereotype. In their specific analyses, controlling for impression management altered the group differences observed for Agreeableness, illustrating that response-presentation adjustment may affect some self-reported trait comparisons but not necessarily others. This finding does not establish that impression management accounts for the EMS differences observed in the present study. Indeed, in the present sensitivity analysis, the multivariate contribution of the EPQ-R Lie score was not statistically significant, and the principal effects of incarceration status, sex, and their interaction remained statistically significant and substantively unchanged. The measured response-presentation tendency therefore did not account for the principal multivariate findings. Nevertheless, the EPQ-R Lie scale cannot capture every form of defensiveness, limited insight, item interpretation, or context-dependent responding, and these possibilities cannot be excluded entirely.

The psychopathy literature provides a related but more specific perspective. Chakhssi et al. (2014) found that psychopathy-related characteristics among offenders with personality disorders were associated with selected EMSs—particularly Mistrust/Abuse and Insufficient Self-Control, together with lower Subjugation—rather than with generalized elevations across all schemas. Moreover, EMSs did not predict institutional violence in that study. Such selective associations are theoretically plausible because psychopathy comprises distinguishable interpersonal, affective, lifestyle, and antisocial features (Hare & Neumann, 2008), which may relate differently, or even oppositely, to individual schemas. These findings help contextualize the present results but cannot explain them, because psychopathy was not directly assessed in the current study. EPQ-R Psychoticism is conceptually and psychometrically distinct from psychopathy, and conviction for homicide must not be treated as evidence of psychopathy.

Taken together, the available evidence argues against interpreting EMS scores along a single continuum from psychologically healthy to psychologically disturbed or from low to high criminogenic risk. The present results are more consistent with multiple, sex-contingent configurations of attachment-related vulnerability, negative self-evaluation, self-regulation, interpersonal functioning, and context-dependent responding. Institutional adaptation, differences in how questionnaire items are interpreted, schema coping styles, and unmeasured personality or psychiatric characteristics remain competing possibilities, but the present cross-sectional design cannot determine which, if any, produced the observed differences. Future studies should therefore combine EMS assessment with validated measures of schema modes, personality pathology, psychopathy, psychiatric comorbidity, and offense-proximal functioning using both self-report and independent clinical or file-based information.

### 4.4. Theoretical Implications

The findings have implications not only for Schema Theory but also for broader criminological accounts of severe violent offending. From a schema perspective, the interaction supports a configuration-based rather than severity-based formulation. The meaning of an individual EMS depends on the broader profile in which it occurs and, in the present data, on the combination of sex and incarceration status. EMSs may therefore be more informative when considered as patterns of cognitive–emotional vulnerability than as a single index of global pathology (Young et al., 2003; Rafaeli et al., 2011).

General Strain Theory provides one possible criminological context for the attachment-related findings. The theory proposes that negative relationships, the loss or threatened loss of positively valued stimuli, and exposure to noxious experiences may generate negative affect and increase pressure toward maladaptive coping under certain conditions (Agnew, 1992). The observed differences in Emotional Deprivation and Abandonment, as well as the broader relevance of threat-related schemas such as Mistrust/Abuse, may be conceptually compatible with histories of relational loss, neglect, rejection, or interpersonal threat. However, EMSs are not measures of strain, and the present study did not assess exposure to strain, anger, coping responses, or the temporal sequence connecting these constructs with offending. The findings therefore identify a potential point of integration between Schema Theory and General Strain Theory but do not constitute a test of the latter (Siserman et al., 2022).

The results also require cautious interpretation in relation to the General Theory of Crime, which identifies low self-control as a central source of criminal and analogous behavior (Gottfredson & Hirschi, 1990). If Insufficient Self-Control/Self-Discipline or Entitlement/Grandiosity were treated as direct schema-level equivalents of criminological low self-control, uniformly higher scores might have been expected among participants convicted of homicide. This component of H1 was not supported: the two schemas were comparatively elevated among community participants, with the interaction showing that this pattern was concentrated among men. The finding does not disconfirm self-control theory, because self-reported EMSs are not interchangeable with behavioral low self-control, opportunity, or offense-specific decision processes. It does show that criminogenic propensity cannot be inferred directly from the magnitude of these schema scores and highlights the importance of distinguishing cognitive endorsement from behavior expressed under different environmental and situational conditions (Gyorgy et al., 2023).

Developmental and life-course criminological perspectives provide a third bridge. Schema Theory conceptualizes EMSs as relatively enduring patterns rooted in early unmet emotional needs, whereas developmental taxonomies and life-course theories emphasize heterogeneity in antisocial trajectories and the dynamic influence of accumulating disadvantage, social bonds, transitions, and turning points (Moffitt, 1993; Sampson & Laub, 1997). Considered together, these perspectives suggest that early cognitive–emotional vulnerabilities may interact over time with family adversity, personality development, peer and social environments, situational opportunities, formal sanctions, and later transitions. Such an interactional account is compatible with the sex-specific heterogeneity observed here, but it was not tested directly. In particular, the present data do not establish that gendered developmental experiences, attachment processes, or life-course trajectories produced the subgroup differences. Moreover, the postdictive design cannot determine whether the observed EMSs predated the index offense, changed through intervening experiences, or were affected by conviction and incarceration.

Taken together, the findings support an integrative framework in which EMSs complement rather than replace personality and criminological explanations. Schema Theory may help characterize how unmet emotional needs and relational adversity are represented cognitively and emotionally; personality models describe broader dispositional patterns; General Strain Theory identifies potentially relevant affective pressures and coping processes; self-control theory directs attention to behavioral regulation and opportunity; and developmental/life-course approaches situate these processes within trajectories unfolding across changing social contexts. Future models should test these levels jointly and longitudinally rather than treating schema endorsement, sex, incarceration, personality, or offense status as sufficient explanations in isolation.

### 4.5. Clinical Implications

The findings have potential implications for psychological assessment and treatment planning among individuals convicted of homicide. EMS assessment may provide clinically relevant information about cognitive–emotional vulnerabilities related to interpersonal functioning, emotion regulation, and coping (Young et al., 2003). However, EMS scores should supplement rather than replace psychiatric assessment, structured violence-risk assessment, personality assessment, and individualized case formulation.

The observed interaction indicates that intervention planning should be based on each person’s schema configuration rather than on offender status or sex alone. At the subgroup level, Emotional Deprivation and Abandonment were comparatively elevated among men convicted of homicide, whereas women convicted of homicide displayed a different and comparatively broader pattern across several schema domains. These group-level findings may identify areas warranting clinical exploration, but individual subgroup contrasts varied in their robustness, and the results do not establish treatment needs or treatment efficacy for any particular person. Nor do they justify assigning treatment targets on the basis of sex. Schema Therapy appropriately emphasizes case conceptualization based on the person’s schemas, coping styles, and schema modes rather than on legal or demographic categories alone (Young et al., 2003; Rafaeli et al., 2011).

The findings also highlight the need for caution when interpreting self-report schema assessments in forensic settings. Limited insight, defensive responding, impression management, and institutional context may affect explicit endorsement of maladaptive schemas (Chakhssi et al., 2014; Hurezan et al., 2024; Oettingen et al., 2024). In the present study, adjustment for EPQ-R Lie scores did not materially alter the principal multivariate findings, meaning that the measured response-presentation tendency did not account for them. This result does not exclude other forms of response bias or context-dependent interpretation. Schema assessment should therefore be integrated, where possible, with structured clinical interviews, collateral and file information, behavioral observations, and other assessment methods.

Finally, EMSs should be regarded as one component of a broader biopsychosocial and criminological formulation that includes developmental adversity, personality functioning, psychiatric comorbidity, substance use, social relationships, situational factors, and environmental opportunities or constraints. The present findings support further evaluation of schema-informed assessment in forensic practice; they do not demonstrate that Schema Therapy reduces violence or recidivism.

### 4.6. Strengths and Limitations

The present study has several methodological and conceptual strengths. To our knowledge, it is among the few studies to examine all 18 EMSs in a comparatively large sample of individuals convicted of homicide while simultaneously investigating the independent and interactive effects of incarceration status and sex. The balanced 2 × 2 design provided adequate and comparable subgroup sizes and permitted direct examination of sex-specific group patterns that would probably have been obscured in a predominantly male forensic sample.

The use of MANCOVA enabled the simultaneous analysis of multiple correlated EMSs while controlling for age and educational attainment. Examining the main effects of incarceration status and sex and their interaction within a single multivariate model reduced reliance on a series of disconnected tests and provided a coherent representation of EMS profiles across the four study subgroups. The complete univariate results are reported with effect sizes, confidence intervals, and both unadjusted and familywise Bonferroni-adjusted probability values, increasing transparency regarding the robustness of individual effects. The additional sensitivity analysis incorporating the EPQ-R Lie score also permitted a limited examination of whether measured response-presentation tendencies materially affected the principal multivariate findings.

Several limitations must nevertheless be acknowledged. First, the study was cross-sectional and postdictive: current EMS scores were compared between groups defined by a previously occurring outcome. Because EMSs were assessed after the index offense, conviction, and incarceration, the study cannot determine whether they preceded the offense, contributed to its occurrence, changed following the offense, or were influenced by subsequent experiences and the correctional environment. The findings therefore describe current group differences and must not be interpreted as evidence that particular schemas predict or cause homicidal behavior.

Second, although the sex-balanced design was advantageous for testing the incarceration status × sex interaction, it does not reproduce the sex distribution typically observed among individuals convicted of homicide. Women were purposefully oversampled relative to their prevalence in the broader correctional population to produce four equally sized analytical groups. Consequently, the observed subgroup comparisons should not be interpreted as population-weighted estimates of EMS prevalence among individuals convicted of homicide. The findings are informative regarding differences between the four study subgroups but cannot be generalized directly to the overall sex composition of homicide-offender populations.

Third, the focal EMS variables were derived from a single self-report instrument administered at one assessment point. Self-reported schema endorsement may be influenced by limited insight, defensive responding, impression management, social desirability, or context-dependent response strategies, particularly in correctional settings. This concern is especially relevant when interpreting the comparatively lower endorsement of several EMSs among incarcerated participants.

Response-presentation tendencies were, however, directly assessed using the EPQ-R Lie scale as part of the broader assessment battery. In the sensitivity analysis, the multivariate contribution of the EPQ-R Lie score was not statistically significant, and adjustment for this score did not materially alter the multivariate effects of incarceration status, sex, or their interaction. These results provide a limited indication that the principal findings were not attributable solely to variation in the measured response-presentation tendency. Nevertheless, this analysis does not constitute a comprehensive correction for common-method variance or all potential forms of defensive responding. The EPQ-R Lie scale captures only one dimension of response style, and both the EMS scores and the Lie score were obtained through self-report within the same broader assessment protocol. Cognitive defenses, institutional adaptation, limited insight, and other forms of response distortion may therefore remain relevant. Future research should combine schema self-report measures with clinician-rated assessments, structured diagnostic interviews, behavioral observations, validated multimethod measures of response bias, and collateral information obtained from correctional and medical records.

Fourth, the community participants were required to have no prior criminal record, previous criminal conviction, or history of incarceration; thus, the comparison group was not defined merely by the absence of a homicide conviction. However, community participants were not systematically screened through structured diagnostic interviews or medical-record reviews. The community group should therefore not be interpreted as a psychiatrically screened or diagnostically homogeneous control group. Unassessed psychiatric conditions, personality pathology, or problematic substance use may have influenced EMS endorsement within this group.

Fifth, incarceration status represents a composite grouping variable that combines several differences that cannot be separated within the present design. The incarcerated and community groups differed not only in homicide-conviction status but also in exposure to criminal proceedings, institutionalization, restricted autonomy, and the interpersonal and social demands of the correctional environment. Consequently, the observed differences cannot be attributed uniquely either to offending history or to incarceration itself.

Sixth, participants were recruited within a single national and correctional context. The incarcerated participants were drawn from several Romanian correctional institutions, whereas the community participants were recruited through university centers, online announcements, and social media platforms. These sampling procedures may limit generalizability to other cultural contexts, legal systems, correctional environments, homicide subtypes, and community populations. Replication should include probability-based or carefully matched community samples and forensic samples recruited from multiple jurisdictions.

Finally, although the broader research protocol included dimensional personality and analytical-reasoning measures, the primary model focused on EMSs and did not incorporate structured assessments of childhood trauma, attachment, schema modes, personality disorders, psychopathy, psychiatric diagnoses, substance-use disorders, or emotion regulation. Psychopathy was not directly assessed, and the EPQ-R Psychoticism dimension must not be interpreted as equivalent to psychopathy or as evidence of a psychotic disorder. The present study therefore cannot determine whether EMSs overlap with, are explained by, or provide incremental information beyond these constructs. Future studies should assess these variables directly and examine their independent and combined contributions within integrated explanatory models.

Despite these limitations, the study provides evidence that EMS profiles among individuals convicted of homicide are selective, heterogeneous, and contingent on sex. The findings support Schema Theory as one framework for investigating cognitive–emotional functioning in forensic populations while demonstrating the need to integrate schema-based accounts with broader developmental, personality, situational, and criminological models.

### 4.7. Future Research

Longitudinal studies are needed to establish the temporal ordering of EMSs, developmental adversity, personality development, offending, conviction, and incarceration. Prospective designs would help determine whether particular schema configurations precede severe violent behavior, change following major life events, or interact with social bonds and turning points across the life course.

Future research should investigate the mechanisms underlying the sex-specific and incarceration-specific patterns identified here. Studies should include measures of impression management, defensiveness, broad and maladaptive personality traits, psychopathy, schema modes, coping styles, emotion regulation, exposure to strain, anger, behavioral self-control, and situational opportunity. Such designs could test whether the observed EMS profiles retain explanatory or clinical value when established psychological and criminological constructs are modeled simultaneously. Explanations based on gendered socialization, attachment, or differential developmental experiences should likewise be tested directly rather than inferred from group membership.

Research should also integrate childhood maltreatment, attachment, psychiatric comorbidity, substance use, and official offending history within multivariate and developmental models. In particular, prospective mediation and moderation analyses could examine whether EMSs represent one mechanism through which relational adversity becomes associated with later coping, interpersonal conflict, or aggression, while preserving the distinction between cognitive–emotional vulnerability and criminal behavior itself.

Finally, replication in larger and more diverse forensic samples is essential. Cross-cultural studies, comparisons across homicide subtypes and different violent-offender groups, and studies with adequately represented female forensic samples would clarify the generalizability and specificity of the present findings. Confirmatory studies should prespecify their primary contrasts and multiplicity-control strategy. Multimethod assessment and repeated measurement across different stages of incarceration would also help distinguish stable schema differences from context-dependent reporting or institutional effects.

## 5. Conclusions

The present study examined current Early Maladaptive Schema (EMS) profiles among incarcerated individuals convicted of homicide and community participants while simultaneously investigating the independent and interactive effects of incarceration status and sex. The principal finding was the significant incarceration status × sex interaction, indicating that the association between incarceration status and EMS configuration differed substantially between women and men. This interaction qualified the aggregate main effects and demonstrated that neither incarceration status nor sex was associated with a uniform schema profile across all four study subgroups.

After correction across the 18 EMS outcomes, incarcerated participants reported higher aggregate scores on Emotional Deprivation and Abandonment, whereas community participants obtained higher scores on several schemas involving impaired autonomy, negative self-evaluation, emotional overcontrol, impaired limits, and self-regulation. These differences were particularly pronounced among men. Mistrust/Abuse did not remain a significant aggregate incarceration-status effect after the familywise correction and should therefore not be considered part of the robust overall status profile. Accordingly, the hypothesis predicting higher Disconnection/Rejection and Impaired Limits schemas among individuals convicted of homicide was only partially supported: the findings supported selected attachment-related differences but contradicted the predicted direction for Entitlement/Grandiosity and Insufficient Self-Control/Self-Discipline.

The results demonstrate that EMS profiles within forensic populations are selective and heterogeneous and cannot be represented adequately by a simple continuum of greater or lesser schema pathology. However, because the study was cross-sectional and postdictive, the observed differences cannot establish whether EMSs preceded homicidal behavior, contributed to its occurrence, changed following the offense, or were influenced by conviction, incarceration, response-presentation tendencies, or other unmeasured characteristics. Explanations involving developmental adversity, attachment, gendered socialization, personality pathology, psychopathy, or institutional adaptation therefore remain hypotheses requiring direct investigation.

From a clinical perspective, EMS assessment may contribute useful information to a broader individualized formulation of cognitive–emotional functioning among people convicted of homicide. Nevertheless, self-reported EMS scores should not be interpreted as direct indicators of violent propensity, criminogenic risk, psychiatric diagnosis, or treatment need. They should be considered alongside structured clinical and violence-risk assessments, personality and psychiatric evaluation, behavioral observations, and collateral or file-based information. The present findings do not demonstrate the effectiveness of Schema Therapy for reducing violence or recidivism but support further evaluation of schema-informed assessment within comprehensive, multimethod forensic research and practice.

Overall, Schema Theory provides one potentially valuable framework for examining cognitive–emotional functioning in forensic populations, but it should complement rather than replace developmental, personality, situational, and criminological explanations. Longitudinal and multimethod studies are needed to determine the temporal origins, incremental explanatory value, and clinical relevance of the sex-contingent EMS configurations identified in the present study.

## Figures and Tables

**Table 1:** Internal consistency of the YSQ-S3: Cronbach’s alpha coefficients for schema domains.

Schema Domain	Cronbach’s α
Disconnection and Rejection	0.92
Impaired Autonomy and Performance	0.90
Impaired Limits	0.86
Other-Directedness	0.88
Overvigilance and Inhibition	0.91
Total scale (18 schemas)	0.95

Note. YSQ-S3 = Young Schema Questionnaire–Short Form 3. Cronbach’s α coefficients are based on standardized items.

**Table 2:** Demographic characteristics of incarcerated and community samples.

Characteristic	Incarcerated (n = 246)	Community (n = 246)	Total (N = 492)
Age, years, M (SD)	37.33 (9.94)	30.96 (10.42)	34.14 (10.66)
Educational level, years, M (SD)	10.93 (1.82)	11.63 (2.13)	11.28 (2.01)
Male, n (%)	123 (50.0)	123 (50.0)	246 (50.0)
Female, n (%)	123 (50.0)	123 (50.0)	246 (50.0)

Note. Values are presented as mean (M) and standard deviation (SD) for the continuous variables (age and educational level), and as frequency (n) with percentage (%) for the categorical variable (sex).

**Table 3:** Forensic, offense-related, sentencing, and criminal-history characteristics of the incarcerated sample.

Characteristic	Total Sample (N = 246)	Men (n = 123)	Women (n = 123)
**Age at assessment, M (SD), years**	37.3 (9.9)	38.0 (9.3)	36.7 (10.5)
**Educational attainment, M, years**	10.9	10.9	10.9
**Age at index offense, M (SD), years**	32.6 (10.0)	33.4 (9.6)	31.7 (10.3)
**Functional classification of the index offense, n (%)**			
First-degree murder	132 (53.7%)	70 (56.9%)	62 (50.4%)
Second-degree murder	75 (30.5%)	36 (29.3%)	39 (31.7%)
Manslaughter/assault resulting in death	32 (13.0%)	14 (11.4%)	18 (14.6%)
Vehicular homicide/negligent killing	7 (2.8%)	3 (2.4%)	4 (3.3%)
**Victim–offender relationship, n (%)**			
Intimate partner or former partner	97 (39.4%)	29 (23.6%)	68 (55.3%)
Other family member	48 (19.5%)	18 (14.6%)	30 (24.4%)
Acquaintance	70 (28.5%)	51 (41.5%)	19 (15.4%)
Stranger	31 (12.6%)	25 (20.3%)	6 (4.9%)
**Number of victims, n (%)**			
One victim	231 (93.9%)	114 (92.7%)	117 (95.1%)
Two victims	11 (4.5%)	7 (5.7%)	4 (3.3%)
Three victims	4 (1.6%)	2 (1.6%)	2 (1.6%)
**Sentence characteristics**			
Fixed-term sentence, n (%)	229 (93.1%)	114 (92.7%)	115 (93.5%)
Life sentence, n (%)	17 (6.9%)	9 (7.3%)	8 (6.5%)
Fixed-term sentence length, M, years	16.6	17.2	15.9
Time served at assessment, M (SD), years	3.6 (3.2)	3.3 (3.1)	3.8 (3.3)
**Criminal history, n (%)**			
No recorded conviction before the index offense	246 (100.0%)	123 (100.0%)	123 (100.0%)
One or more convictions before the index offense	0 (0.0%)	0 (0.0%)	0 (0.0%)
No recorded violent conviction before the index offense	246 (100.0%)	123 (100.0%)	123 (100.0%)
One or more violent convictions before the index offense	0 (0.0%)	0 (0.0%)	0 (0.0%)
Age at first conviction	Not applicable	Not applicable	Not applicable

Note. Values are presented as M (SD) or n (%). Percentages were calculated within columns and may not sum to 100% because of rounding. Index-offense categories are reported using jurisdiction-neutral functional labels to facilitate interpretation across legal systems. Fixed-term sentence length was calculated only for participants serving determinate sentences. Prior convictions refer to convictions predating the index offense. Age at first conviction was not applicable because no prior convictions were recorded. M = mean; SD = standard deviation.

**Table 4:** Multivariate effects of age, educational attainment, incarceration status, sex, and their interaction on Early Maladaptive Schemas (MANCOVA).

Effect	Pillai’s Trace	*F*(18, 469)	*p*	Partial η^2^
Age	0.062	1.71	0.035	0.062
Educational attainment	0.065	1.81	0.022	0.065
Incarceration status	0.417	18.65	<0.001	0.417
Sex	0.446	20.94	<0.001	0.446
Incarceration status × Sex	0.411	18.21	<0.001	0.411

Note. Multivariate effects are reported using Pillai’s Trace. Age and educational attainment were included as covariates.

**Table 5:** Adjusted estimated marginal means of Early Maladaptive Schemas according to incarceration status.

Early Maladaptive Schema	Incarcerated M (SE)	Community M (SE)	*p*
Emotional Deprivation	14.45 (0.45)	9.95 (0.45)	<0.001
Abandonment	13.91 (0.42)	11.10 (0.42)	<0.001
Mistrust/Abuse	15.40 (0.40)	14.18 (0.40)	0.036
Social Isolation	14.13 (0.42)	13.63 (0.42)	0.419
Defectiveness/Shame	10.39 (0.41)	13.53 (0.41)	<0.001
Failure	12.72 (0.44)	16.63 (0.44)	<0.001
Dependence/Incompetence	12.93 (0.47)	18.99 (0.47)	<0.001
Vulnerability to Harm or Illness	11.12 (0.53)	19.00 (0.53)	<0.001
Enmeshment/Undeveloped Self	12.41 (0.53)	21.73 (0.53)	<0.001
Subjugation	12.52 (0.56)	23.23 (0.56)	<0.001
Self-Sacrifice	19.12 (0.57)	29.26 (0.57)	<0.001
Emotional Inhibition	15.43 (0.64)	28.20 (0.64)	<0.001
Unrelenting Standards	16.36 (0.66)	30.79 (0.66)	<0.001
Entitlement/Grandiosity	14.72 (0.77)	29.99 (0.77)	<0.001
Insufficient Self-Control/Self-Discipline	13.61 (0.82)	29.56 (0.82)	<0.001
Approval Seeking	40.72 (0.90)	46.02 (0.90)	<0.001
Negativity/Pessimism	31.20 (0.79)	37.58 (0.79)	<0.001
Punitiveness	43.79 (0.79)	45.95 (0.79)	0.061

Note. Values represent estimated marginal means adjusted for age and educational attainment. *p* values are based on Bonferroni-adjusted pairwise comparisons between groups. SE = standard error.

## Data Availability

The data presented in this study are available from the corresponding author upon request. The data are not publicly available due to ethical restrictions.

## Supplementary Materials

The following supporting information can be downloaded at: https://www.mdpi.com/article/10.3390/bs16091535/s1, Table S1: Univariate ANCOVA Results for Age, Educational Attainment, Incarceration Status, Sex, and the Incarceration Status × Sex Interaction Across the 18 Early Maladaptive Schemas.
