# Supplementary material for: A Comparative Study of Early Maladaptive Schema Profiles in Individuals Convicted of Homicide and Individuals from the Community: The Moderating Role of Sex
Source: Behav Sci (Basel). 2026 Aug 31;16(9):1535. doi: 10.3390/bs16091535 (PMC13603524; doi:10.3390/bs16091535)
Supplement: Supplementary file 1 [file behavsci-16-01535-s001.zip › behavsci-4469017-supplementary.pdf]

Supplementary Table S1

Univariate ANCOVA Results for Age, Educational Attainment, Incarceration Status, Sex, and the Incarceration Status × Sex Interaction Across the 18 Early Maladaptive Schemas

Panel A. Age

Early Maladaptive Schema	F	p	p_B	ηp^2	95% CI for ηp^2
Emotional Deprivation	6.02	.015	.261	.012	[.000, .039]
Abandonment	1.73	.189	1.000	.004	[.000, .022]
Mistrust/Abuse	1.18	.278	1.000	.002	[.000, .019]
Social Isolation	0.21	.643	1.000	.000	[.000, .012]
Defectiveness/Shame	3.36	.067	1.000	.007	[.000, .029]
Failure	3.86	.050	.902	.008	[.000, .031]
Dependence/Incompetence	3.63	.057	1.000	.007	[.000, .030]
Vulnerability to Harm or Illness	6.92	.009	.158	.014	[.001, .042]
Enmeshment/Undeveloped Self	12.08	< .001	**.010**	.024	[.005, .058]
Subjugation	10.69	.001	**.021**	.022	[.003, .053]
Self-Sacrifice	9.91	.002	**.031**	.020	[.003, .051]
Emotional Inhibition	16.34	< .001	**.001**	.033	[.009, .069]
Unrelenting Standards	10.20	.001	**.027**	.021	[.003, .052]
Entitlement/Grandiosity	7.07	.008	.146	.014	[.001, .042]
Insufficient Self-Control/Self-Discipline	6.18	.013	.238	.013	[.000, .039]
Approval Seeking	3.79	.052	.940	.008	[.000, .030]
Negativity/Pessimism	1.33	.249	1.000	.003	[.000, .020]
Punitiveness	0.95	.331	1.000	.002	[.000, .017]

Panel B. Educational attainment

Early Maladaptive Schema	*F*	*p*	p_B	ηp^2	95% CI for ηp^2
Emotional Deprivation	0.00	.972	1.000	.000	[.000, .000]
Abandonment	0.13	.718	1.000	.000	[.000, .010]
Mistrust/Abuse	0.01	.917	1.000	.000	[.000, .005]
Social Isolation	5.22	.023	.409	.011	[.000, .036]
Defectiveness/Shame	12.62	< .001	**.008**	.025	[.005, .059]
Failure	17.93	< .001	**< .001**	.036	[.010, .074]
Dependence/Incompetence	16.16	< .001	**.001**	.032	[.009, .069]
Vulnerability to Harm or Illness	19.55	< .001	**< .001**	.039	[.012, .078]
Enmeshment/Undeveloped Self	15.95	< .001	**.001**	.032	[.008, .068]
Subjugation	19.79	< .001	**< .001**	.039	[.012, .078]
Self-Sacrifice	13.54	< .001	**.005**	.027	[.006, .062]
Emotional Inhibition	17.50	< .001	**< .001**	.035	[.010, .072]
Unrelenting Standards	16.86	< .001	**< .001**	.034	[.009, .071]
Entitlement/Grandiosity	21.46	< .001	**< .001**	.042	[.014, .083]
Insufficient Self-Control/Self-Discipline	21.44	< .001	**< .001**	.042	[.014, .082]
Approval Seeking	6.88	.009	.162	.014	[.001, .042]
Negativity/Pessimism	11.17	< .001	**.016**	.022	[.004, .055]
Punitiveness	3.68	.056	1.000	.008	[.000, .030]

Panel C. Incarceration status

Early Maladaptive Schema	*F*	*p*	p_B	ηp²	95% CI for ηp²
Emotional Deprivation	47.03	< .001	**< .001**	.088	[.046, .139]
Abandonment	20.87	< .001	**< .001**	.041	[.014, .081]
Mistrust/Abuse	4.42	.036	.649	.009	[.000, .033]
Social Isolation	0.65	.419	1.000	.001	[.000, .016]
Defectiveness/Shame	27.95	< .001	**< .001**	.054	[.022, .098]
Failure	36.94	< .001	**< .001**	.071	[.033, .118]
Dependence/Incompetence	78.98	< .001	**< .001**	.140	[.088, .197]
Vulnerability to Harm or Illness	104.32	< .001	**< .001**	.177	[.120, .237]
Enmeshment/Undeveloped Self	144.43	< .001	**< .001**	.229	[.168, .291]
Subjugation	172.01	< .001	**< .001**	.261	[.199, .323]
Self-Sacrifice	147.36	< .001	**< .001**	.233	[.172, .294]
Emotional Inhibition	187.33	< .001	**< .001**	.278	[.215, .340]
Unrelenting Standards	222.13	< .001	**< .001**	.314	[.250, .375]
Entitlement/Grandiosity	186.93	< .001	**< .001**	.278	[.215, .340]
Insufficient Self-Control/Self-Discipline	176.00	< .001	**< .001**	.266	[.203, .328]
Approval Seeking	16.45	< .001	**.001**	.033	[.009, .070]
Negativity/Pessimism	30.60	< .001	**< .001**	.059	[.025, .104]
Punitiveness	3.53	.061	1.000	.007	[.000, .029]

Panel D. Sex

Early Maladaptive Schema	*F*	*p*	p_B	ηp²	95% CI for ηp²
Emotional Deprivation	48.89	< .001	**< .001**	.091	[.049, .143]
Abandonment	44.05	< .001	**< .001**	.083	[.042, .133]
Mistrust/Abuse	30.08	< .001	**< .001**	.058	[.024, .103]
Social Isolation	0.78	.377	1.000	.002	[.000, .016]
Defectiveness/Shame	50.10	< .001	**< .001**	.093	[.050, .145]
Failure	23.50	< .001	**< .001**	.046	[.017, .088]
Dependence/Incompetence	32.50	< .001	**< .001**	.063	[.027, .108]
Vulnerability to Harm or Illness	58.84	< .001	**< .001**	.108	[.062, .162]
Enmeshment/Undeveloped Self	84.50	< .001	**< .001**	.148	[.095, .206]
Subjugation	127.54	< .001	**< .001**	.208	[.148, .269]
Self-Sacrifice	56.36	< .001	**< .001**	.104	[.058, .157]
Emotional Inhibition	138.57	< .001	**< .001**	.222	[.161, .283]
Unrelenting Standards	138.78	< .001	**< .001**	.222	[.162, .284]
Entitlement/Grandiosity	106.93	< .001	**< .001**	.180	[.123, .240]
Insufficient Self-Control/Self-Discipline	120.18	< .001	**< .001**	.198	[.140, .259]
Approval Seeking	0.94	.332	1.000	.002	[.000, .017]
Negativity/Pessimism	15.70	< .001	**.002**	.031	[.008, .068]
Punitiveness	5.86	.016	.285	.012	[.000, .038]

Panel E. Incarceration status × Sex interaction

**Early Maladaptive Schema**	***F***	***p***	p_B	**ηp²**	**95% CI for ηp²**
Emotional Deprivation	9.86	.002	**.032**	.020	[.003, .051]
Abandonment	0.62	.431	1.000	.001	[.000, .015]
Mistrust/Abuse	2.45	.118	1.000	.005	[.000, .025]
Social Isolation	42.07	< .001	**< .001**	.080	[.040, .129]
Defectiveness/Shame	72.36	< .001	**< .001**	.130	[.079, .186]
Failure	105.55	< .001	**< .001**	.178	[.122, .238]
Dependence/Incompetence	148.05	< .001	**< .001**	.234	[.172, .295]
Vulnerability to Harm or Illness	180.00	< .001	**< .001**	.270	[.207, .332]
Enmeshment/Undeveloped Self	228.53	< .001	**< .001**	.320	[.256, .381]
Subjugation	230.37	< .001	**< .001**	.322	[.258, .383]
Self-Sacrifice	213.37	< .001	**< .001**	.305	[.241, .366]
Emotional Inhibition	255.32	< .001	**< .001**	.344	[.281, .405]
Unrelenting Standards	265.47	< .001	**< .001**	.353	[.290, .413]
Entitlement/Grandiosity	191.97	< .001	**< .001**	.283	[.220, .345]
Insufficient Self-Control/Self-Discipline	214.74	< .001	**< .001**	.306	[.243, .368]
Approval Seeking	71.89	< .001	**< .001**	.129	[.079, .185]
Negativity/Pessimism	117.18	< .001	**< .001**	.194	[.136, .255]
Punitiveness	39.69	< .001	**< .001**	.075	[.037, .124]

Note. Each row reports an outcome-specific ANCOVA from the primary 2 × 2 MANCOVA. All models included incarceration status and sex as fixed factors, their interaction, and age and educational attainment as covariates. All F tests have df = 1, 486. p is the unadjusted probability value. pB is the Bonferroni-adjusted probability value across the 18 EMS outcomes within the relevant effect family. The Bonferroni option specified for estimated marginal means in SPSS applies to those pairwise comparisons and does not adjust the outcome-specific omnibus ANCOVA tests; pB is reported here as an additional familywise sensitivity correction. Confidence intervals for ηp² were obtained by inversion of the noncentral F distribution. Bold pB values indicate statistical significance at α = .05. EMS = Early Maladaptive Schema; CI = confidence interval; ηp² = partial eta squared.
